# Parylene Double-Layer Coated Screen-Printed Carbon Electrode for Label-Free and Reagentless Capacitive Aptasensing of Gliadin

**DOI:** 10.1021/acssensors.4c00875

**Published:** 2024-07-10

**Authors:** Chun-Ning Tsai, Chin-Yun Lee, Hsien-Yeh Chen, Bo-Chuan Hsieh

**Affiliations:** † Department of Biomechatronics Engineering, National Taiwan University, Taipei 10617, Taiwan; ‡ Department of Chemical Engineering, National Taiwan University, Taipei 10617, Taiwan

**Keywords:** aptamer, insulation layer, Parylene C, Parylene AM, gluten

## Abstract

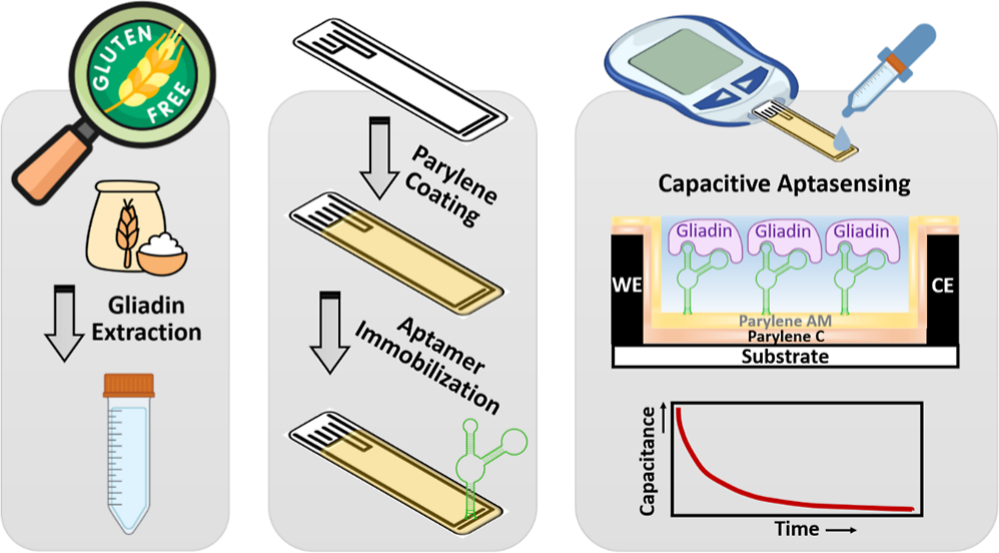

Celiac patients are required to strictly adhere to a gluten-free diet because even trace amounts of gluten can damage their small intestine and leading to serious complications. Despite increased awareness, gluten can still be present in products due to cross-contamination or hidden ingredients, making regular monitoring essential. With the goal of guaranteeing food safety for consuming labeled gluten-free products, a capacitive aptasensor was constructed to target gliadin, the main allergic gluten protein for celiac disease. The success of capacitive aptasensing was primarily realized by coating a Parylene double-layer (1000 nm Parylene C at the bottom with 400 nm Parylene AM on top) on the electrode surface to ensure both high insulation quality and abundant reactive amino functionalities. Under the optimal concentration of aptamer (5 μM) used for immobilization, a strong linear relationship exists between the amount of gliadin (0.01–1.0 mg/mL) and the corresponding Δ*C* response (total capacitance decrease during a 20 min monitoring period after sample introduction), with an *R*^2^ of 0.9843. The detection limit is 0.007 mg/mL (S/*N* > 5), equivalent to 0.014 mg/mL (14 ppm) of gluten content. Spike recovery tests identified this system is free from interferences in corn and cassava flour matrices. The analytical results of 24 commercial wheat flour samples correlated well with a gliadin ELISA assay (*R*^2^ = 0.9754). The proposed label-free and reagentless capacitive aptasensor offers advantages of simplicity, cost-effectiveness, ease of production, and speediness, making it a promising tool for verifying products labeled as gluten-free (gluten content <20 ppm).

Gluten is a group of proteins exist in wheat, barley, rye, and related products. It provides elasticity to dough, helping it rise and maintain its shape during baking. Gluten is commonly found in many staple foods, such as bread, pasta, and cereals. Celiac disease is an autoimmune disorder characterized by an intolerance to gluten. When people with celiac disease ingest gluten, their immune system reacts by attacking the small intestine, resulting in inflammation and harm to the lining of the intestine. This damage can result in various digestive and nutritional problems. The main approach to managing celiac disease is a lifelong, strict gluten-free diet (a gluten-free product is defined as having less than 20 ppm of gluten). Removing gluten from the diet helps alleviate symptoms and allows the intestinal lining to heal. Individuals with celiac disease must be vigilant about avoiding not only obvious sources of gluten but also hidden sources in processed foods, medications, and other products.^1−4^ Consequently, a fast, low-cost, and reliable screening method is highly demanded to verify products labeled as gluten-free for celiac patients.

Gliadin, the major alcohol-soluble component among wheat gluten, is unable to be completely digested by intestinal enzymes. The remaining 33-mer gliadin peptide is currently considered to be the main trigger for celiac disease.^5^ Therefore, researchers often develop analytical tools for gluten in foods by targeting gliadin or its immunogenic peptide. For example, the detection can be carried out using immunoassays such as enzyme-linked immunosorbent assays (ELISA) and lateral flow devices (LFD), known for their simplicity, cost-effectiveness, and time-saving. However, these methods may encounter false negatives (ELISA) or provide nonquantitative results (LFD). Although genomics-based alternatives such as quantitative polymerase chain reaction can achieve higher specificity and sensitivity for gliadin compared to ELISA and LFD, they are still time-consuming and require well-trained operators. Proteomics-based approaches, such as liquid chromatography–mass spectrometry, are promising and powerful tools for the reproducible and precise identification of gliadin peptides at lower concentration levels, but they necessitate a complex operational procedure, expensive equipment, and a long analysis time.^6−9^

Over the past decade, aptamer-based biosensors (aptasensors) have garnered significant attention across diverse applications in clinical diagnostics, environmental monitoring, food safety, and bioanalytical research due to a multitude of advantages. Aptamers are novel biorecognition elements that can be selected to bind specifically to a wide range of target molecules of interest, including ions, toxins, proteins, viruses, bacteria, and even whole cells. This ensures accurate and sensitive detection, as well as ease of customization. Aptamers can be designed to have minimal cross-reactivity with similar molecules, reducing the chances of false positives in detection assays. Their rapid target recognition ability also makes them suitable for real-time screening uses. The ease of versatile modification, such as the incorporation of functional groups (e.g., thiol, amino, hydroxyl) and conjugation of labels (e.g., fluorescent dyes, enzymes), enhances adaptability to different experimental setups. Additionally, aptamers exhibit stability under a variety of conditions, including changes in temperature and pH, contributing to their robustness for use in diverse environments. Furthermore, aptamers can be synthesized relatively easily and inexpensively, making the production of aptasensors cost-effective compared to other biorecognition elements like antibodies.^10−12^ Recently, an aptamer sequence called Gli4 was successfully selected against the immunotoxic 33-mer gliadin peptide. It was applied in a competitive electrochemical magneto assay that enabled gluten quantification down to 0.5 ppm, with no cross-reactivity to the matrices of nongluten containing grains.^13^ Later, a simpler architecture of a competitive electrochemical assay was developed with the Gli4 aptamer, achieving a lower detection limit of 0.38 ppm for gluten.^14^ However, despite meeting the requirements for gluten-free product monitoring in terms of both selectivity and sensitivity, those system designs still rely on the strategies of enzyme labeling and the addition of electrochemical active redox reagents. The lack of simplicity did hamper its implementation as a point of care testing device.

Capacitive biosensors achieve real-time and ultrasensitive detection by measuring minute alterations in the properties of the dielectric layer at the electrode–electrolyte interface when specific target molecules bind to the biorecognition elements immobilized on the sensing surface. The capacitance *C* of this dielectric layer between two electrodes can be determined using the basic equation as follows
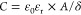
1where ε_0_ represents the dielectric constant in vacuum (8.85 pF/m), ε_r_ stands for its dielectric constant, *A* indicates its area, and δ is the thickness of dielectric layer.

This nonfaradaic biosensing technique often operates in a label-free and reagentless mode, requiring only simple readout electronics. This simplicity is beneficial for miniaturization and incorporation into portable devices, offering a promising solution for low-cost, convenient, and on-site diagnostics in point of care testing applications.^15,16^ Various capacitive aptasensing platforms have been developed and successfully applied to detect small molecules such as organophosphorus pesticides^17^ and bisphenol A,^18^ proteins like thrombin^19^ and human epidermal growth factor receptor,^20^ as well as cells such as *Escherichia coli*(21) and lung carcinoma cells.^22^ The fabrication of an insulation layer covering on the electrode surface is considered to be the most important issue for the success of capacitive aptasensing. This layer must be well-insulated and hole-free to avoid charge leakage to the electrode, preventing dramatic fluctuations in the baseline capacitance level. Besides, the layer should possess reactive functionalities for the subsequent chemical conjugation of the biorecognition elements. To maintain good sensitivity, this layer also needs to be as thin as possible.^23^

Parylene is the trade name referring to a family of polymers known as poly(*p*-xylylene). These polymers are typically produced through the chemical vapor deposition (CVD) process, free from solvents, catalysts, and plasticizers. During CVD, the granular dimer precursor (di-*p*-xylylene) vaporizes and forms a uniform, hole-free pure polymeric coating onto the substrate, creating a conformal layer with customizable thickness ranging from tens of nanometers to tens of micrometers. Parylene coatings offer several desirable properties, including high chemical resistance, low moisture permeability, and electrical insulation. The use of Parylene as an insulation layer is advantageous because it provides a protective barrier without altering the dimensions or features of the underlying substrates. This conformal coating ensures that even complex and intricate structures are adequately insulated, contributing to the reliability and longevity of electronic devices. To date, there are over 10 commercially accessible variants of Parylene. The unsubstituted, monochloro-substituted, and dichloro-substituted versions, named Parylene N, Parylene C, and Parylene D, respectively, are the most commonly used industrial coatings. Among them, Parylene C is an FDA-approved class IV biocompatible polymer due to its excellent water and gas barrier properties and has been widely applied to implantable devices. Other versions of functionalized Parylene, such as Parylene A (monoamino-substituted), Parylene AM (monoaminomethyl-substituted), and Parylene H (monoaldehyde-substituted) coatings, can also serve as chemical anchors, providing reactive functional groups for the immobilization of biomolecules. These coatings have been successfully utilized for biomedical and biosensing purposes.^24−27^

Since the deposition of Parylene is a low-cost, well-established industrial manufacturing technique that enables finely controlled conformal coating along with mass production capability, it can also offer a series of advantages such as being ultrathin, chemically inert, providing high electrical insulation, and possessing reactive functionalities. Therefore, it may be an ideal insulation layer for constructing capacitive biosensors and further implementation as a point of care testing device. To the best of our knowledge, this study represents the first attempt to apply Parylene C (bottom layer) and Parylene AM (top layer) as the functionalized insulation double-layer for capacitive aptasensing. The double-layer was coated onto the surface of a screen-printed carbon electrode (SPCE), followed by immobilizing the 5′-NH_2_-modified Gli4 aptamer using the cross-linker glutaraldehyde, and ultimately for the detection of gliadin in commercial wheat flours (Figure 1). These analytical results were further compared with those obtained from a commercial gliadin ELISA assay. Although false negatives may occur with the ELISA assay when gliadins are denatured during certain food processing procedures, it remains the most recommended method for providing acceptable results regarding the raw materials used in gluten-free food production by the AOAC International.^7,9^ Therefore, the AOAC-approved ELISA assay was chosen as the reference method in the current study. Additionally, interferences from other nongluten containing flour matrices were also evaluated.

**Figure 1:**
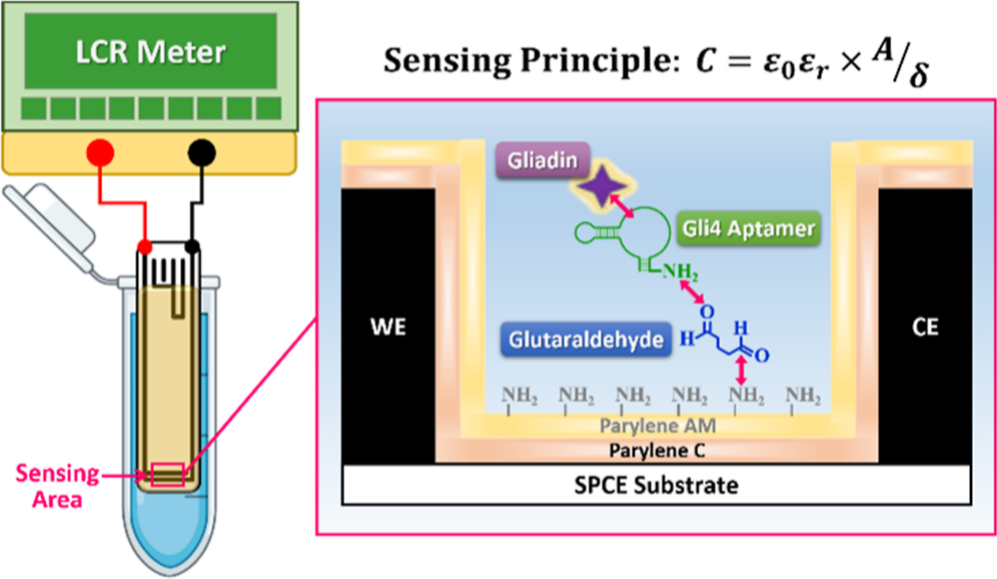
Schematic illustration of the capacitive aptasensor for gliadin analysis. Parylene C and Parylene AM were sequentially coated onto an SPCE via CVD, followed by immobilization of the 5′-NH_2_-modified Gli4 aptamer onto the sensing area using glutaraldehyde. The prepared SPCE was connected to the LCR meter probes and immersed in the testing solution. The change in capacitance resulting from the affinity binding between aptamer and gliadin was monitored and calculated for quantification.

## Experimental Section

### Reagents and Samples

Glutaraldehyde (25% aqueous solution), methanol, sodium dihydrogen phosphate, and sodium hydrogen phosphate were sourced from Nacalai Tesque (Kyoto, Japan). Fluorescein isothiocyanate (FITC), gliadin (from wheat), glycine, hydrochloric acid, magnesium chloride, sodium chloride, and tris(hydroxymethyl) aminomethane were procured from Sigma-Aldrich (St. Louis, USA). All reagents were of analytical grade and required no additional purification. Commercial wheat flours, corn flour, and cassava flour were obtained from local supermarkets. The RIDASCREEN Gliadin Assay (R7021) was provided by *R*-Biopharm (Darmstadt, Germany). The Gli4 aptamer sequence has a total length of 40 nucleotides: 5′-CCA GTC TCC CGT TTA CCG CGC CTA CAC ATG TCT GAA TGC C-3′. The aptamers, including 3′-fluorescein amidite-labeled (3′-FAM-labeled) and/or 5′-NH_2_-modified ones, were synthesized and purified by PURIGO Biotechnology (Taipei, Taiwan).

### Parylene Coating

The Parylene C coating service was provided by Taiwan Innova Efficiency (Taipei, Taiwan). The Parylene AM coating was achieved using a self-designed CVD polymerization system.^28^ The CVD conditions involved initially sublimating the Parylene AM dimer precursor at 100–120 °C under reduced pressure (0.2–0.3 Torr). Subsequently, it was transferred into the pyrolysis zone, kept at 670 °C, through a constant argon flow (5 cc/min). The material vapor finally reached a temperature-controlled sample holder (15 °C), where it spontaneously polymerized onto the surface of SPCEs (from Delta Electronics, Taipei, Taiwan). The surface topography of the Parylene-coated SPCE and the coating thickness were acquired by a three-dimensional profile confocal laser microscope (VK-9500, Keyence Corporation, Osaka, Japan).

### Aptamer Immobilization

Aliquots of the aptamer solution were diluted with a 50 mM Tris–HCl buffer, pH 7.4, containing 250 mM NaCl and 5 mM MgCl_2_ to the desired concentration. The mixture was heated at 98 °C for 5 min, cooled to 4 °C for 5 min, and then conditioned at 25 °C for 30 min to ensure the proper folding of its specific three-dimensional conformation. Onto the sensing area of the Parylene-coated SPCE, 40 μL of a 5% glutaraldehyde solution was initially dropped and allowed to react for 90 min. The electrode was then washed with double-distilled water. Subsequently, it was treated with 40 μL of the aforementioned aptamer solution and kept in a moisture-saturated box at 4 °C overnight to implement the cross-linking reaction between Parylene AM and the 5′-NH_2_-modified aptamer through glutaraldehyde. After washing out the unbound aptamers, 40 μL of a 100 mM glycine solution was added for 90 min to block the free aldehyde groups of glutaraldehyde, preventing nonspecific binding of other proteins during the real sample aptasensing. To examine the success of aptamer immobilization, a fluorescence microscope (Zeiss Axio Vert.A1, M&T Optics, Taipei, Taiwan) was used for observation.

### Capacitance and Dissipation Factor Measurements

The Parylene-coated SPCE or the subsequently aptamer-immobilized SPCE was connected to the LCR meter probes (LCR-6300, Good Will Instrument, Taipei, Taiwan) and immersed in an Eppendorf tube containing 2 mL of phosphate-buffered saline (PBS) + solution (0.1 M PBS, pH 7.0, containing 1 mM MgCl_2_). The capacitance and dissipation factor (*D* value) were simultaneously examined using the series equivalent circuit with a measurement frequency of 10 kHz and a bias voltage of 0.5 V. The lower the *D* value, the less power loss when AC is applied through the capacitor, indicating better insulation quality of the Parylene coating.

### Capacitive Aptasensing of Gliadin

To ensure the insulation quality of aptamer-immobilized SPCE for the success of capacitive aptasensing, a 30 min capacitance measurement was initially conducted in a 2 mL PBS + solution. Upon observing a stable baseline capacitance level, 0.2 mL of PBS + solution was extracted and replaced with 0.2 mL of gliadin sample solution (0.01–1.0 mg/mL, dissolved in 70% methanol). The total decrease in capacitive response resulting from the affinity binding between the aptamer and gliadin during the immediately subsequent 20 min measurement was monitored and calculated for further regression analyses.

### Analysis of Gliadin in Real Samples

In a test tube, a total of 1 g of commercial flour sample was mixed with 10 mL of 70% methanol and ultrasonicated for 20 min. The extraction mixture was then centrifuged at 10,000*g* for 15 min. The supernatant solution from the wheat flour sample was used to replace 10% of the volume of PBS + solution for capacitive aptasensing. This supernatant was directly used for gliadin ELISA assay quantification. The supernatant solutions from corn flour and cassava flour were spiked with aliquots of the gliadin standard solution to achieve the desired concentration, and then used to replace 10% of the volume of PBS + solution for recovery tests by capacitive aptasensing.

## Results and Discussion

### Characterization of Parylene Coating

**Figure 2:**
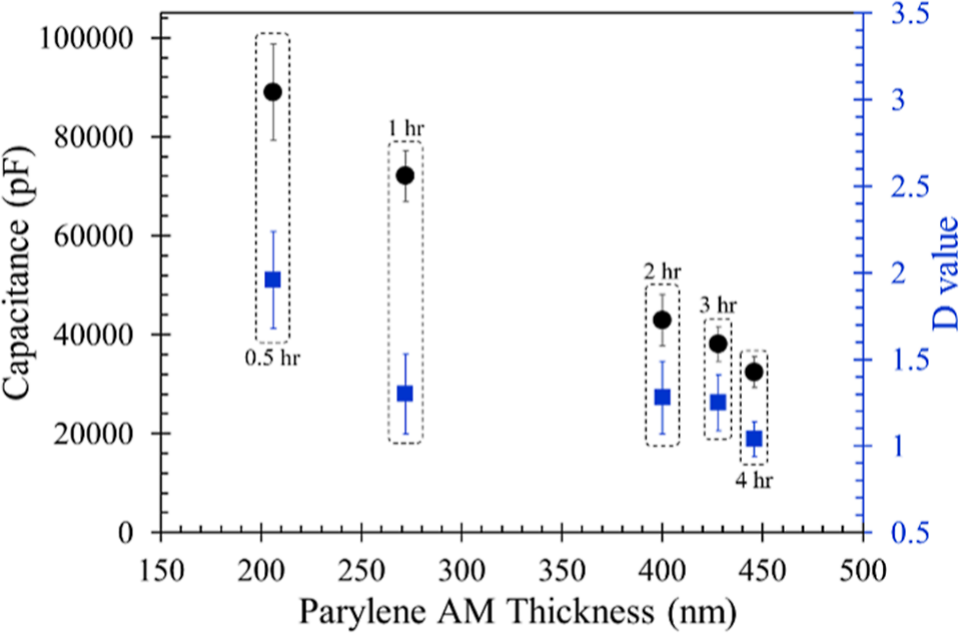
Thickness, capacitance (circles), and *D* value (squares) of Parylene AM coatings with CVD time from 0.5 to 4 h (*N* = 5). Capacitances and *D* values were recorded by an LCR meter at 0.5 V, 10 kHz for 10 min in the PBS + solution, and all consecutive data points were used for statistical analysis. The error bars indicate standard deviation of duplicate experiments.

Our initial attempt was to coat a single layer of Parylene AM to assess whether the simplest design could achieve the desired insulation quality, along with its inherent reactive functionalities for the subsequent aptamer immobilization. As depicted in Figure 2, the thickness of Parylene AM clearly increased with CVD time from 0.5 h (206 nm) to 2 h (400 nm). Subsequently, the rate of thickness increase appeared to slow down, with thicknesses of 428 nm for 3 h coating and 446 nm for 4 h coating. This finding indicates that the deposition of Parylene AM through CVD can hardly create a thicker layer over 500 nm within a few hours. Furthermore, there is a tendency for capacitance to decrease with the increase in Parylene AM thickness. This occurs because the covering of thicker Parylene AM at the electrode–electrolyte interface leads to a gradual increase in the thickness of the dielectric layer, as stated in eq 1. However, all conditions of Parylene AM coating exhibited poor insulation quality, not only as indicated by *D* values exceeding 1.0, but also by the capacitance responses remaining in the tens of nF range with significant deviations. This phenomenon may be attributed to the Parylene AM coating not providing sufficient compactness to cover the rough, undulatory electrode surface, thus failing to achieve effective electrical insulation. This deficiency leads to noticeable charge leakage, consequently resulting in the formation of faradaic current at the electrode–electrolyte interface and causing significant fluctuations in the baseline capacitance level.

**Figure 3:**
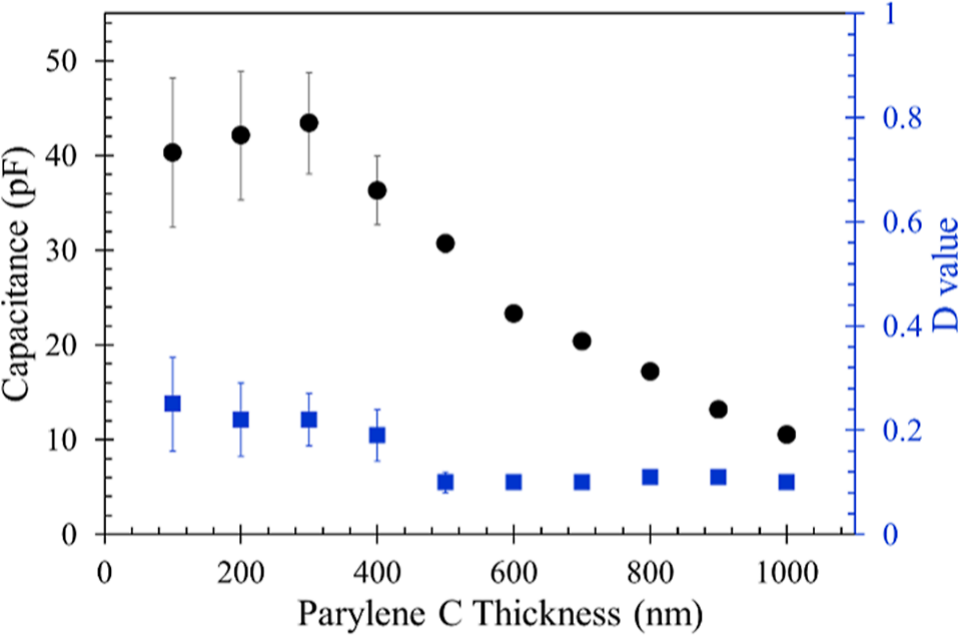
Capacitance (circles), and *D* value (squares) of Parylene C coatings with thickness from 100 to 1000 nm (*N* = 5). All experimental conditions were the same as described in Figure 2. The error bars indicate standard deviation of duplicate experiments.

A compromising approach was then implemented by coating Parylene C to guarantee thorough insulation in advance. As can be seen from Figure 3, Parylene C indeed provided better insulation quality compared to Parylene AM, as evidenced by the *D* values of all coating thicknesses being below 0.3, and the capacitance responses falling within the tens of pF range. Considering both lower *D* values and smaller deviations in capacitance, Parylene C coating thicknesses ranging from 500 to 1000 nm were selected as the bottom layer for the subsequent Parylene AM top layer coating experiments.

**Figure 4:**
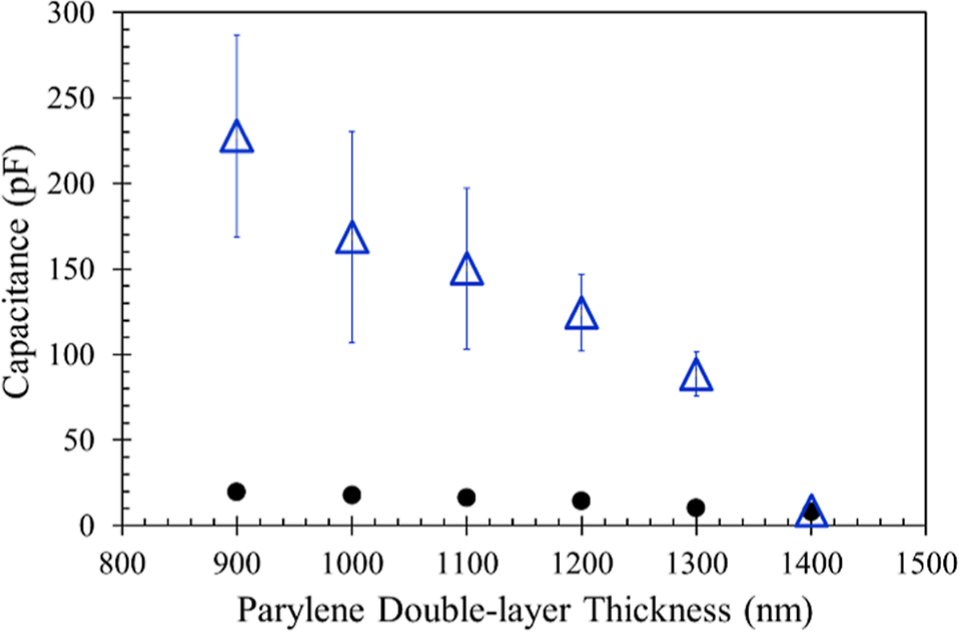
Effect of overnight immersion (12 h) in PBS + solution on capacitance measurement for Parylene double-layer coatings (*N* = 5). The double-layer comprised of a Parylene C thickness ranging from 500 to 1000 nm as the bottom layer, and a fixed Parylene AM thickness of 400 nm on top. Circles: without overnight immersion; triangles: with overnight immersion. All experimental conditions were the same as described in Figure 2. The error bars indicate standard deviation of duplicate experiments.

With a fixed Parylene AM coating thickness of 400 nm on top of Parylene C (400 ± 23.4 nm, *N* = 5), the measured capacitance (see the circles in Figure 4) further declined compared to Figure 3, which is consistent with the description in eq 1. To verify long-term water resistance, these Parylene double-layer coated electrodes were immersed in the buffer solution overnight (12 h). However, undesirable fluctuations in capacitance and elevated capacitance levels were observed for all groups following immersion treatment, except for the 1400 nm thickness group (see the triangles in Figure 4). This circumstance proves that only the Parylene double-layer coating thicker than 1400 nm can provide preferable insulation quality for the subsequent aptamer immobilization and capacitive gliadin detection in an aqueous environment.

Although self-assembled monolayers (SAMs) are the most commonly used insulating strategy for capacitive biosensing applications,^17,19,20,22^ they still have limitations that need to be overcome. For instance, they are sensitive to experimental conditions, and gradual degradation may destroy the insulation quality during biosensing. The type of headgroup of SAMs is limited, restricting their versatility beyond metallic electrode surfaces. Additionally, the formation of a uniform coverage is time-consuming, often requiring an overnight process. In contrast, Parylene is chemically inert with high electrical insulation and can be deposited onto all kinds of underlying substrates within a few hours, making it superior for constructing capacitive biosensing platforms over SAMs.

**Figure 5:**
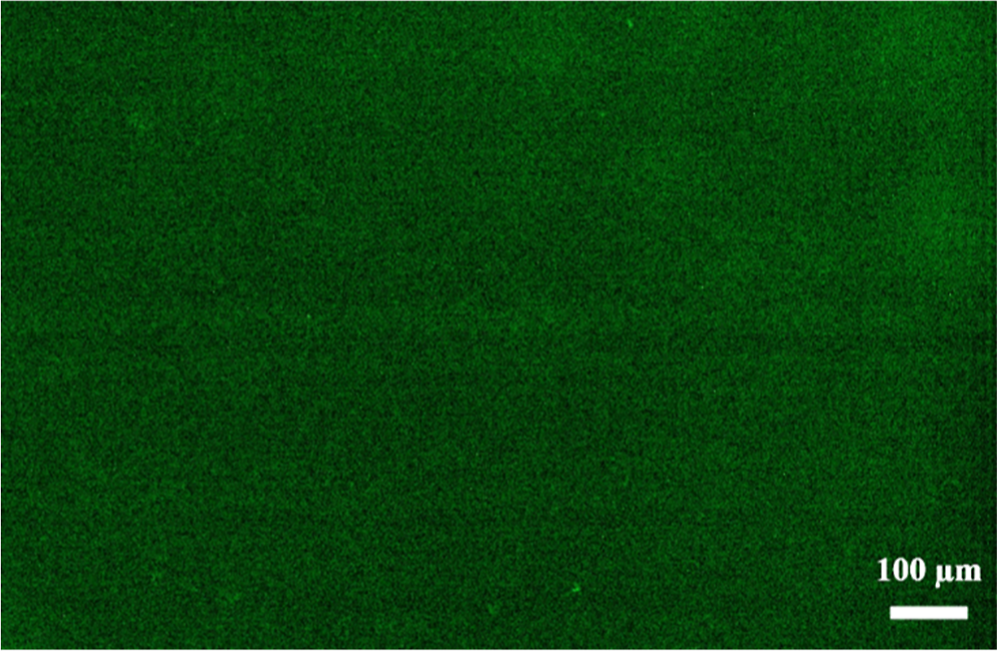
Fluorescence image of the 1400 nm Parylene double-layer coating with FITC dye.

To assess the reactivity of inherent amino functional groups on the Parylene double-layer surface, a green fluorescent dye, FITC, was employed to specifically label its free amino groups. Figure 5 reveals a high density of FITC response, suggesting abundant reactive amino functionalities on the surface of the Parylene double-layer. The fluorescence coverage was estimated to be 72.2 ± 4.1% (*N* = 3). This finding is beneficial for the subsequent chemical conjugation of biorecognition elements.

**Figure 6:**
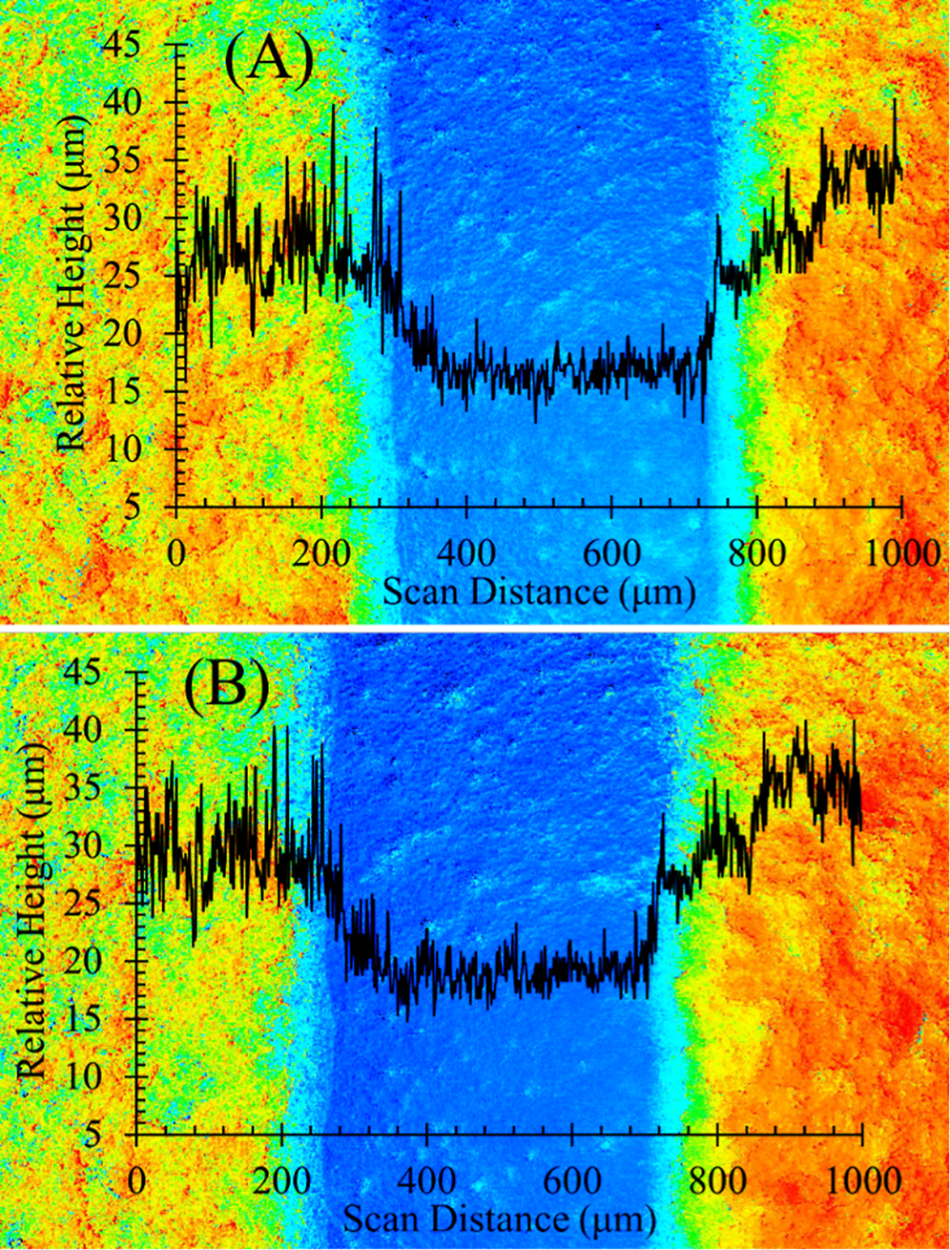
Top view of surface topography: (A) bare SPCE, and (B) SPCE coated with 1400 nm Parylene double-layer. The black curve illustrates the relative height of the scanned area. The central blue region indicates the underlying SPCE substrate, while the adjacent areas in orange, yellow, and green represent the carbon paste electrode pair.

For ultrasensitive capacitive sensing, it is essential to preserve the cavity space between an electrode pair as much as possible after Parylene coating. This is because the majority of electric field lines concentrate in this region. Even a minute change in dielectric properties within the cavity space can result in the most sensitive capacitive response. As depicted in Figure 6A, the surface profile of a bare SPCE displays a height difference of approximately 15 μm between the lateral carbon paste electrodes and the central underlying substrate. This height difference remains almost unchanged when a 1400 nm Parylene double-layer is applied afterward (Figure 6B). This phenomenon is in accordance with the described feature of Parylene coating through CVD, which is its ability to form a uniform and conformal layer without altering the dimensions of the underlying substrate. However, there is still plenty of cavity space remaining while covering on the Parylene double-layer, which means the ultrasensitive capacitive aptasensing may be implemented as expected.

### Characterization of Aptamer Immobilization

**Figure 7:**
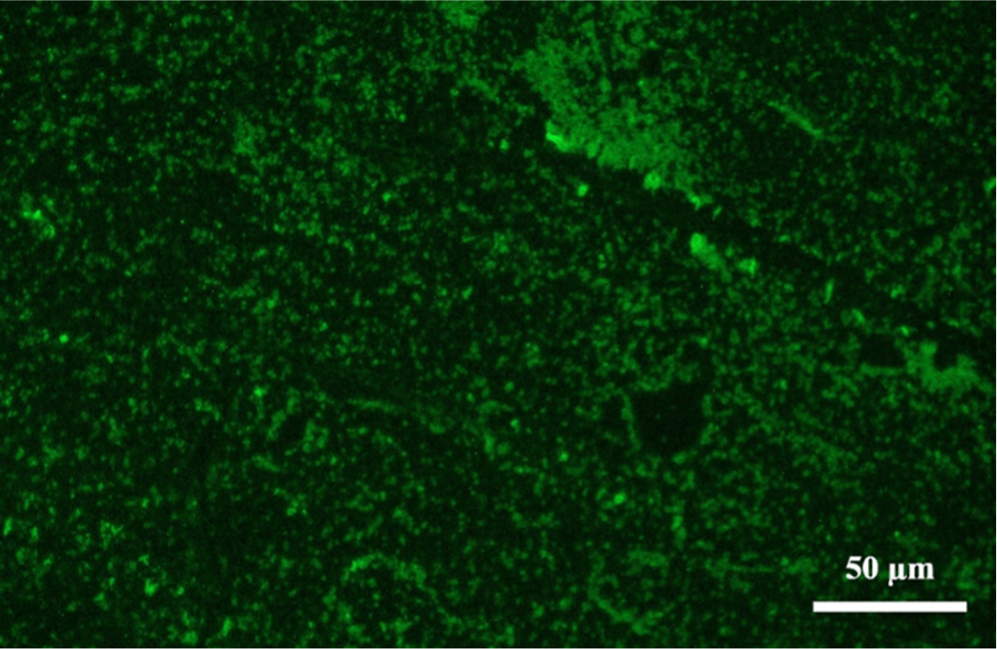
Fluorescence image of the 1400 nm Parylene double-layer coating after immobilizing 5 μM of the 3′-FAM-labeled and 5′-NH_2_-modified Gli4 aptamer through glutaraldehyde.

To confirm the success of aptamer immobilization, 5 μM of 3′-FAM-labeled and 5′-NH_2_-modified Gli4 aptamer was cross-linked onto the surface of Parylene double-layer using glutaraldehyde. The fluorescence microscopic observation in Figure 7 clearly reveals the high intensity of green fluorescence emitted by the successfully immobilized aptamer. The fluorescence coverage was determined to be 58.2 ± 4.8% (*N* = 3).

### Capacitive Aptasensing of Gliadin

**Figure 8:**
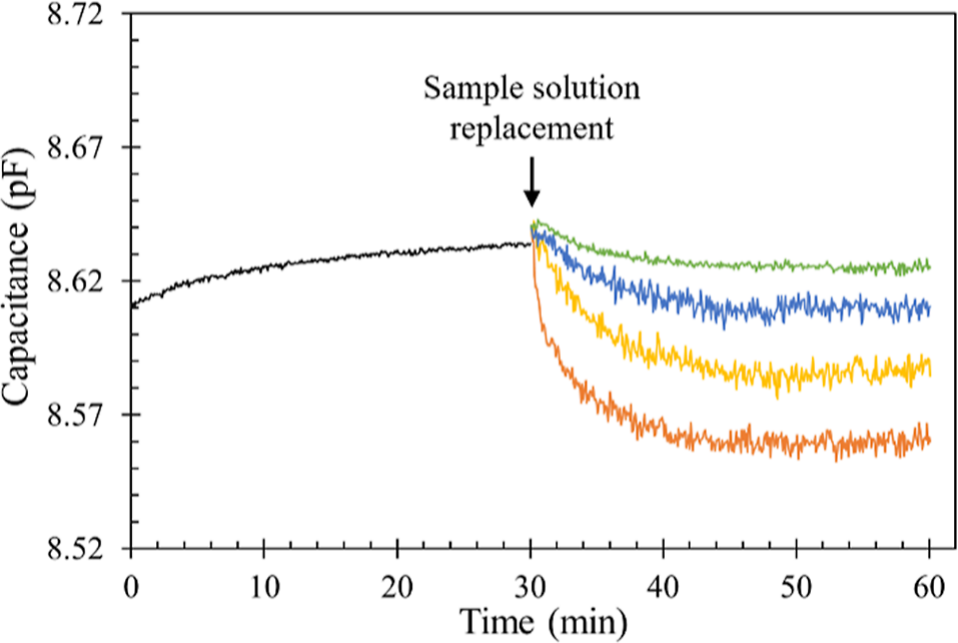
Typical capacitive response of gliadin aptasensing. The black curve represents the baseline in a 2 mL PBS + solution. The other overlaid curves depict the replacement of 0.2 mL of PBS + solution with 0.2 mL of gliadin sample solution. Replacement with 70% ethanol (the solvent of gliadin, shown in green), 0.01 mg/mL gliadin (blue), 0.1 mg/mL gliadin (yellow), and 1.0 mg/mL gliadin (orange). Capacitance was recorded by an LCR meter at 0.5 V, 10 kHz for 60 min.

Before introducing the gliadin sample into the test solution, a 30 min capacitance baseline measurement was conducted in a 2 mL PBS + solution (black curve in Figure 8). For a well-insulated aptamer-immobilized sensing electrode, a stable capacitance level below 10 pF can be observed, with a standard deviation lower than 0.004 pF (0.0024–0.0039 pF, *N* = 5) for consecutive data points during the last 10 min of monitoring. The flow disturbance attributed to the action of 10% sample solution volume replacement immediately led to an increase in capacitance. Following the replacement with 70% ethanol (the solvent of gliadin, green curve), the capacitance returned to a stable level within the subsequent 10 min (the standard deviation for consecutive data points during the period of 10–20 min after sample replacement ranged from 0.0021 to 0.0034 pF, *N* = 5). This suggests that the steady-state dielectric layer thickness can be achieved within a 10 min period after the operational disturbance. Other overlaid curves, depicting the presence of gliadin (in blue, yellow, and orange), further illustrate the dynamic binding behavior of Gli4 aptamer–gliadin interactions. The addition of more gliadin corresponds to a greater capacitive decrease, attributable to the synergistic effect of forming a thickened dielectric layer and replacing high dielectric constant water molecules (ε_r_ = 80) with lower dielectric constant protein molecules (ε_r_ = 20), such as gliadin, as described in eq 1. Additionally, the gradual decrease in capacitance seems to stabilize after 20 min of sample introduction, the total decrease in capacitance during the 20 min consecutive monitoring was therefore used for gliadin quantification.

To achieve optimal analytical performance in capacitive aptasensing, we investigated the immobilized dosage of the aptamer ranging from 1 to 10 μM. As shown in Table 1, a strong linear relationship was observed between the capacitive response and gliadin concentration within the dynamic range of 0.01–1.0 mg/mL for all aptamer dosages used during immobilization. The sensitivity of gliadin aptasensing can be clearly enhanced with an elevated aptamer dosage from 1 to 5 μM (see the slopes in Table 1). However, it worsens when the dosage is increased to 10 μM. This occurrence may result from the denser immobilization of aptamers on the surface of the Parylene double-layer coated electrode. When gliadin molecules are captured by the immobilized aptamers, the conformational change in the aptamers results in steric hindrance to neighboring aptamers, creating insufficient space to conjugate with target gliadin molecules. Therefore, 5 μM aptamer appears to be an optimal concentration for the present work. The relative standard deviations were less than 10.5% for all concentration points (*N* = 5), and gliadin concentrations lower than 0.007 mg/mL (7 ppm) could be determined with an S/N ratio higher than 5. Since approximately 50% of wheat gluten proteins consist of monomeric gliadins,^29^ our LOD value for gluten should be calculated by multiplying it by a factor of 2,^30^ resulting in 14 ppm. Although this LOD for gluten is higher than that obtained by the competitive electrochemical magneto assay^13^ and the competitive electrochemical assay,^14^ both developed with the same Gli4 aptamer, it still fulfills the European legislated threshold of 20 ppm required for verifying food labeling as gluten-free. The storage stability of the constructed aptasensor was preliminarily tested by storing the aptamer-immobilized SPCEs in PBS + solution at 4 °C. After a one-week storage period, a negligible loss in the capacitive aptasensing response was observed. However, further long-term systematic evaluation is needed.

**Table 1:** Effect of Immobilized Dosage of Gli4 Aptamer on the Calibration Curve of Capacitive Aptasensing of Gliadin

aptamer dosage (μM)	linearity (mg/mL)	slope	*R*^2^
1	0.01–1.0	0.0810	0.9532
3	0.01–1.0	0.0900	0.9910
5	0.01–1.0	0.1071	0.9843
10	0.01–1.0	0.1012	0.9636

Although other label-free and reagentless biosensing alternatives, such as the quartz crystal microbalance-based immunosensor,^31^ the localized surface plasmon resonance-based immunological assay,^32^ and the gold nanoparticle aggregation-based colorimetric aptasensor,^33^ have demonstrated more sensitive performance in gliadin detection than the proposed capacitive aptasensing platform, our method remains superior in terms of cost-effectiveness, robustness under environmental conditions, and simplicity in the design of readout electronics. These advantages make it particularly suitable for developing point-of-care testing devices.

### Analysis of Gliadin in Real Samples

**Figure 9:**
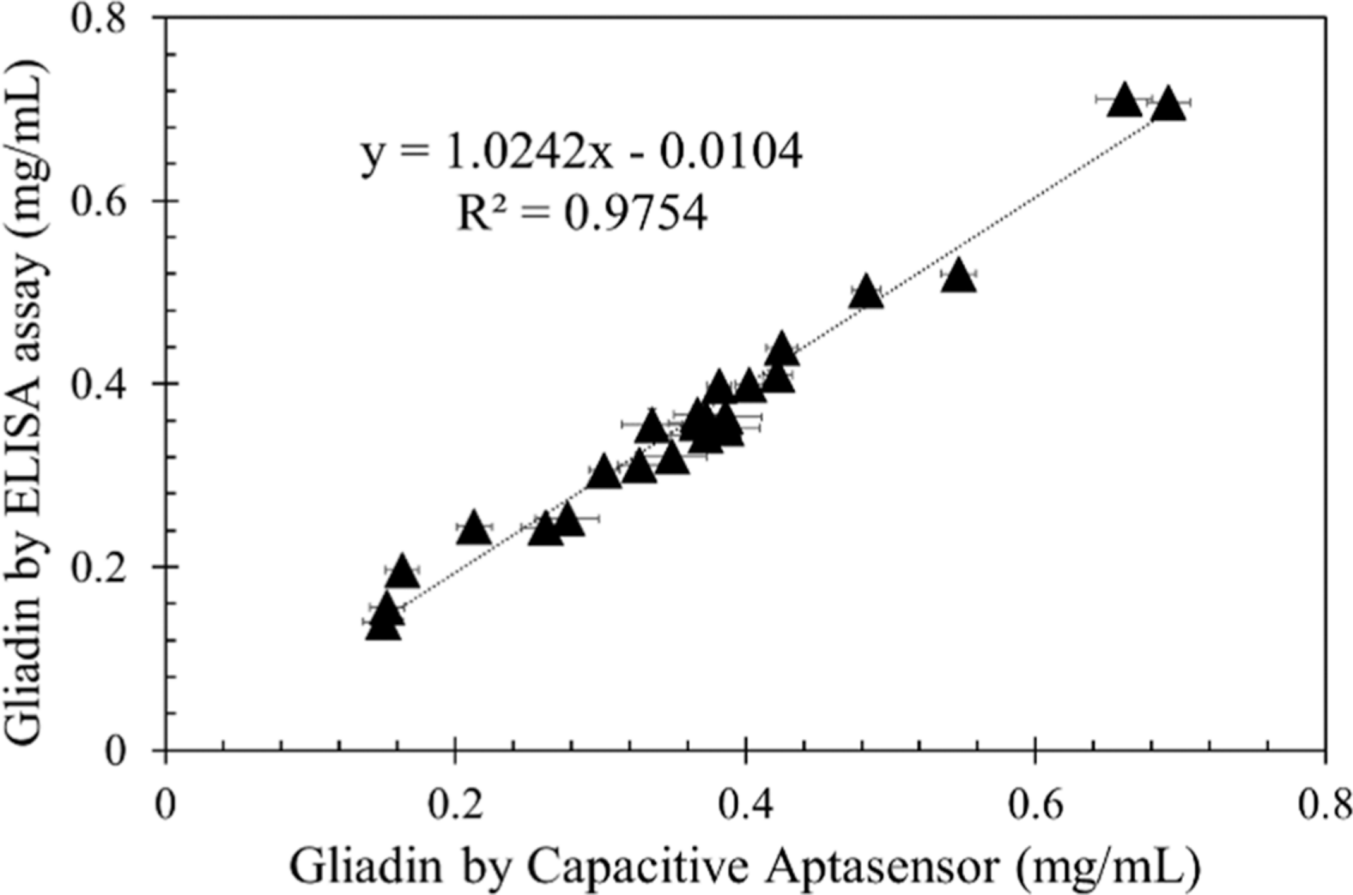
Comparison of the analytical results of 24 commercial wheat flour samples determined by the proposed capacitive aptasensor (*N* = 3) and by a gliadin ELISA assay (*N* = 3). The error bars indicate standard deviation of duplicate experiments.

Twenty-four commercial wheat flour samples were employed to evaluate the reliability of the proposed capacitive aptasensor by comparing its analytical results with those obtained from a gliadin ELISA assay. By introducing the supernatant extract into the test solution, the corresponding capacitance decrease was recorded and then interpolated into the calibration curve obtained with gliadin standards to determine the gliadin content in real samples. As depicted in Figure 9, a strong positive linear correlation (*R*^2^ = 0.9754) was observed, indicating that the proposed method offers accurate detection of gliadin in real gluten-containing samples. Moreover, two commonly used gluten-free grain samples were spiked with varying concentrations of gliadin. The recovery values, ranging from 93.9 to 107.1% (Table 2), suggest that the developed system is interference-free from the matrices of corn and cassava flours, further proving its potential for practical uses.

**Table 2:** Recovery Tests of Spiked Corn and Cassava Flour Matrices (*N* = 3)

sample	gliadin spiked (mg/mL)	gliadin found (mg/mL)	recovery (%)
corn flour	0.1	0.1071	107.1
	1.0	1.0287	102.9
cassava flour	0.1	0.0939	93.9
	1.0	0.9756	97.6

## Conclusions

The feasibility of utilizing an ultrathin Parylene double-layer as the functionalized insulation conformal coating for capacitive aptasensing of gliadin has been successfully identified in the present study. This label-free, reagentless, nonfaradaic biosensing approach can be realized through a very simple experimental setup, making it readily available to construct a portable device by incorporating a commercial integrated circuit chip designed for switched capacitance measurement. This advancement is beneficial for performing low-cost, convenient, and real-time field analysis of gluten-containing substances in the near future. Although our LOD value (14 ppm for gluten) still fulfills the European legislated threshold for gluten-free product verification, it is expected to be further lowered by improving the fabrication process of the electrode pair to obtain both a higher aspect ratio and a flattened undulatory electrode surface, which is currently under investigation.

## References

[ref1] Biesiekierski J. R. What is gluten?. J. Gastroenterol. Hepatol. 2017, 32, 78–81. 10.1111/jgh.13703. 28244676

[ref2] Caio G.; Volta U.; Sapone A.; Leffler D. A.; De Giorgio R.; Catassi C.; Fasano A. Celiac disease: a comprehensive current review. BMC Med. 2019, 17, 142 10.1186/s12916-019-1380-z. 31331324 PMC6647104

[ref3] Aljada B.; Zohni A.; El-Matary W. The Gluten-Free Diet for Celiac Disease and Beyond. Nutrients 2021, 13, 3993 10.3390/nu13113993. 34836247 PMC8625243

[ref4] Guennouni M.; Admou B.; El khoudri N.; Bourrhouat A.; Zogaam L. G.; Elmoumou L.; Hilali A. Gluten contamination in labelled gluten-free, naturally gluten-free and meals in food services in low-middle- and high-income countries: a systematic review and meta-analysis. Br. J. Nutr. 2022, 127, 1528–1542. 10.1017/S0007114521002488. 34753529

[ref5] Woldemariam K. Y.; Yuan J.; Wan Z.; Yu Q.; Cao Y.; Mao H.; Liu Y.; Wang J.; Li H.; Sun B. Celiac Disease and Immunogenic Wheat Gluten Peptides and the Association of Gliadin Peptides with HLA DQ2 and HLA DQ8. Food Rev. Int. 2022, 38, 1553–1576. 10.1080/87559129.2021.1907755.

[ref6] Scherf K. A.; Poms R. E. Recent developments in analytical methods for tracing gluten. J. Cereal Sci. 2016, 67, 112–122. 10.1016/j.jcs.2015.08.006.

[ref7] Xhaferaj M.; Alves T. O.; Ferreira M. S. L.; Scherf K. A. Recent progress in analytical method development to ensure the safety of gluten-free foods for celiac disease patients. J. Cereal Sci. 2020, 96, 103114 10.1016/j.jcs.2020.103114.

[ref8] Ribeiro M.; de Sousa T.; Sabença C.; Poeta P.; Bagulho A. S.; Igrejas G. Advances in quantification and analysis of the celiac related immunogenic potential of gluten. Rev. Food Sci. Food Saf. 2021, 20, 4278–4298. 10.1111/1541-4337.12828. 34402581

[ref9] Wieser H.; Segura V.; Ruiz-Carnicer Á.; Sousa C.; Comino I. Food Safety and Cross-Contamination of Gluten-Free Products: A Narrative Review. Nutrients 2021, 13, 2244 10.3390/nu13072244. 34210037 PMC8308338

[ref10] Mishra G. K.; Sharma V.; Mishra R. K. Electrochemical Aptasensors for Food and Environmental Safeguarding: A Review. Biosensors 2018, 8, 28 10.3390/bios8020028. 29570679 PMC6022872

[ref11] Kaur H.; Shorie M. Nanomaterial based aptasensors for clinical and environmental diagnostic applications. Nanoscale Adv. 2019, 1, 2123–2138. 10.1039/C9NA00153K. 36131986 PMC9418768

[ref12] Chen W.; Lai Q.; Zhang Y.; Liu Z. Recent Advances in Aptasensors For Rapid and Sensitive Detection of Staphylococcus Aureus. Front. Bioeng. Biotechnol. 2022, 10, 889431 10.3389/fbioe.2022.889431. 35677308 PMC9169243

[ref13] Amaya-González S.; de-los-Santos-Álvarez N.; Miranda-Ordieres A. J.; Lobo-Castañón M. J. Aptamer Binding to Celiac Disease-Triggering Hydrophobic Proteins: A Sensitive Gluten Detection Approach. Anal. Chem. 2014, 86, 2733–2739. 10.1021/ac404151n. 24502317

[ref14] López-López L.; Miranda-Castro R.; de-los-Santos-Álvarez N.; Miranda-Ordieres A. J.; Lobo-Castañón M. J. Disposable electrochemical aptasensor for gluten determination in food. Sens. Actuators, B 2017, 241, 522–527. 10.1016/j.snb.2016.10.112.

[ref15] Weaver S.; Mohammadi M. H.; Nakatsuka N. Aptamer-functionalized capacitive biosensors. Biosens. Bioelectron. 2023, 224, 115014 10.1016/j.bios.2022.115014. 36628826

[ref16] Huang L.; Zhang C.; Ye R.; Yan B.; Zhou X.; Xu W.; Guo J. Capacitive biosensors for label-free and ultrasensitive detection of biomarkers. Talanta 2024, 266, 124951 10.1016/j.talanta.2023.124951. 37487266

[ref17] Zhang J.; Fang X.; Wu J.; Hu Z.; Jiang Y.; Qi H.; Zheng L.; Xuan X. An interdigitated microelectrode based aptasensor for real-time and ultratrace detection of four organophosphorus pesticides. Biosens. Bioelectron. 2020, 150, 111879 10.1016/j.bios.2019.111879. 31767346

[ref18] Cui H.; Cheng C.; Lin X.; Wu J.; Chen J.; Eda S.; Yuan Q. Rapid and sensitive detection of small biomolecule by capacitive sensing and low field AC electrothermal effect. Sens. Actuators, B 2016, 226, 245–253. 10.1016/j.snb.2015.11.129.

[ref19] Chen H.-J.; Chen R. L. C.; Hsieh B.-C.; Hsiao H.-Y.; Kung Y.; Hou Y.-T.; Cheng T.-J. Label-free and reagentless capacitive aptasensor for thrombin. Biosens. Bioelectron. 2019, 131, 53–59. 10.1016/j.bios.2019.02.025. 30826650

[ref20] Qureshi A.; Gurbuz Y.; Niazi J. H. Label-free capacitance based aptasensor platform for the detection of HER2/ErbB2 cancer biomarker in serum. Sens. Actuators, B 2015, 220, 1145–1151. 10.1016/j.snb.2015.06.094.

[ref21] Dua P.; Ren S.; Lee S. W.; Kim J.-K.; Shin H.-S.; Jeong O.-C.; Kim S.; Lee D.-K. Cell-SELEX Based Identification of an RNA Aptamer for Escherichia coli and Its Use in Various Detection Formats. Mol. Cells 2016, 39, 807–813. 10.14348/molcells.2016.0167. 27871171 PMC5125936

[ref22] Nguyen N.-V.; Yang C.-H.; Liu C.-J.; Kuo C.-H.; Wu D.-C.; Jen C.-P. An Aptamer-Based Capacitive Sensing Platform for Specific Detection of Lung Carcinoma Cells in the Microfluidic Chip. Biosensors 2018, 8, 98 10.3390/bios8040098. 30347814 PMC6316635

[ref23] Robin P.; Gerber-Lemaire S. Design and Preparation of Sensing Surfaces for Capacitive Biodetection. Biosensors 2022, 13, 17 10.3390/bios13010017. 36671852 PMC9856139

[ref24] Chen H.-Y.; Lahann J. Designable Biointerfaces Using Vapor-Based Reactive Polymers. Langmuir 2011, 27, 34–48. 10.1021/la101623n. 20590103

[ref25] Kim B. J.; Meng E. Micromachining of Parylene C for bioMEMS. Polym. Adv. Technol. 2016, 27, 564–576. 10.1002/pat.3729.

[ref26] Golda-Cepa M.; Engvall K.; Hakkarainen M.; Kotarba A. Recent progress on parylene C polymer for biomedical applications: A review. Prog. Org. Coat. 2020, 140, 105493 10.1016/j.porgcoat.2019.105493.

[ref27] Coelho B. J.; Pinto J. V.; Martins J.; Rovisco A.; Barquinha P.; Fortunato E.; Baptista P. V.; Martins R.; Igreja R. Parylene C as a Multipurpose Material for Electronics and Microfluidics. Polymers 2023, 15, 2277 10.3390/polym15102277. 37242852 PMC10224276

[ref28] Chen H.-Y.; Lin T.-J.; Tsai M.-Y.; Su C.-T.; Yuan R.-H.; Hsieh C.-C.; Yang Y.-J.; Hsu C.-C.; Hsiao H.-M.; Hsu Y.-C. Vapor-based tri-functional coatings. Chem. Commun. 2013, 49, 4531–4533. 10.1039/c3cc41491d. 23575991

[ref29] Wieser H.; Koehler P.; Scherf K. A. Chemistry of wheat gluten proteins: Qualitative composition. Cereal Chem. 2023, 100, 23–35. 10.1002/cche.10572.

[ref30] Gessendorfer B.; Koehler P.; Wieser H. Preparation and characterization of enzymatically hydrolyzed prolamins from wheat, rye, and barley as references for the immunochemical quantitation of partially hydrolyzed gluten. Anal. Bioanal. Chem. 2009, 395, 1721–1728. 10.1007/s00216-009-3080-6. 19763549

[ref31] Funari R.; Terracciano I.; Ventura B. D.; Ricci S.; Cardi T.; D’Agostino N.; Velotta R. Label-Free Detection of Gliadin in Food by Quartz Crystal Microbalance-Based Immunosensor. J. Agric. Food Chem. 2017, 65, 1281–1289. 10.1021/acs.jafc.6b04830. 28121432

[ref32] Bariani G. C.; Zhou L.; Poggesi S.; Manzano M.; Ionescu R. E. Patterning Large-Scale Nanostructured Microarrays on Coverslip for Sensitive Plasmonic Detection of Aqueous Gliadin Traces. Chemosensors 2022, 10, 38 10.3390/chemosensors10020038.

[ref33] Ham S. H.; Kim E.; Han H.; Lee M. G.; Choi Y. J.; Hahn J. A label-free aptamer-based colorimetric biosensor for rapid gliadin detection in foods: a focus on pasta, bread and cookies. Anal. Methods 2024, 16, 449–457. 10.1039/D3AY01695A. 38165727

